# Underlying causes of persistently elevated acute phase reactants in patiens with Familial Mediterranean Fever

**DOI:** 10.1186/1546-0096-13-S1-P139

**Published:** 2015-09-28

**Authors:** R Mercan, B Bitik, R Eren, B Dumludag, A Turan, H Kucuk, MA Ozturk, A Tufan

**Affiliations:** 1 Antakya R&T Hospital, Hatay, Turkey; 2 Ankara R&T Hospital, Rheumatology, Ankara, Turkey; 3 Gazi University, Rheumatology, Ankara, Turkey; 4 Yildirim Beyazit R&T Hospital, Ankara, Turkey

## Background

Erythrocyte sedimentation rate (ESR) and C-reactive protein (CRP) are most commonly employed acute phase reactants in follow up of patients with Familial Mediterranean Fever (FMF). As a rule CRP increases during FMF attacks but it returns to normal values in attack free periods. Persistently elevated acute phase reactants in attack free periods can be occasionally observed in patients with FMF and is suggested to be a risk for the development of amyloidosis. Some authors suggested the use of IL-1 antagonists in such patients to prevent from amyloidosis. However there is no data regarding causes of elevated acute phase reactants in patients with FMF.

## Objectives

In this study we aimed to investigate causes of persistently high ESR and CRP in patients with FMF.

## Methods

Electronic medical records of our well-defined FMF cohort were analyzed. Persistently elevated CRP was defined as more than 2-fold increased values and elevated ESR was defined as > 40 mm/h arbitrarily and these must be evident in all consecutive visits in a year period. There were at least four visits in a year for each patient. Measurements performed during attacks or apparent infections were ignored. A detailed history and physical examination was performed in each patient. All patients were underwent relevant tests according to their clinical evaluation.

## Results

There were 310 patients in cohort and 83 (26.8%) of them was found to have elevated CRP and ESR. Twenty six (31%) patients had spondyloarthritis who fulfilled ASAS criteria for axial spondyloarthritis. In 34 patients (41%) either attacks were very frequent or patients had chronic manifestations of disease (chronic arthritis, myalgia, ascites etc) indicating active FMF. Four patients had inflammatory bowel disease. One patient had Sjögren's syndrome, 1 had scleroderma and 1 had vasculitis. In 16 patients (19.3) any cause couldn't be identified.

## Conclusions

The most common causes of persistently elevated acute phase reactants were found to be active FMF disease, spondyloarthritis and inflammatory bowel disease which could be easily identified with history and simple tests. However in a substantial number of patients any cause couldn't be identified. Therefore, not all patients with increased CRP and ESR are good candidates for IL-1 antagonists. A through history and simple tests can identify most causes of increased acute phase reactants in these individuals.

